# Event-related potentials to task-irrelevant changes in facial expressions

**DOI:** 10.1186/1744-9081-5-30

**Published:** 2009-07-20

**Authors:** Piia Astikainen, Jari K Hietanen

**Affiliations:** 1 Department of Psychology, PO Box 35, 40014 University of Jyväskylä, Jyväskylä, Finland; 2 Human Information Processing Laboratory, Department of Psychology, 33014 University of Tampere, Tampere, Finland

## Abstract

**Background:**

Numerous previous experiments have used oddball paradigm to study change detection. This paradigm is applied here to study change detection of facial expressions in a context which demands abstraction of the emotional expression-related facial features among other changing facial features.

**Methods:**

Event-related potentials (ERPs) were recorded in adult humans engaged in a demanding auditory task. In an oddball paradigm, repeated pictures of faces with a neutral expression ('standard', p = .9) were rarely replaced by pictures with a fearful ('fearful deviant', p = .05) or happy ('happy deviant', p = .05) expression. Importantly, facial identities changed from picture to picture. Thus, change detection required abstraction of facial expression from changes in several low-level visual features.

**Results:**

ERPs to both types of deviants differed from those to standards. At occipital electrode sites, ERPs to deviants were more negative than ERPs to standards at 150–180 ms and 280–320 ms post-stimulus. A positive shift to deviants at fronto-central electrode sites in the analysis window of 130–170 ms post-stimulus was also found. Waveform analysis computed as point-wise comparisons between the amplitudes elicited by standards and deviants revealed that the occipital negativity emerged earlier to happy deviants than to fearful deviants (after 140 ms versus 160 ms post-stimulus, respectively). In turn, the anterior positivity was earlier to fearful deviants than to happy deviants (110 ms versus 120 ms post-stimulus, respectively).

**Conclusion:**

ERP amplitude differences between emotional and neutral expressions indicated pre-attentive change detection of facial expressions among neutral faces. The posterior negative difference at 150–180 ms latency resembled visual mismatch negativity (vMMN) – an index of pre-attentive change detection previously studied only to changes in low-level features in vision. The positive anterior difference in ERPs at 130–170 ms post-stimulus probably indexed pre-attentive attention orienting towards emotionally significant changes. The results show that the human brain can abstract emotion related features of faces while engaged to a demanding task in another sensory modality.

## Background

The ability to detect changes in one's environment is important to survival. Not surprisingly, perhaps, change detection has been shown to work pre-attentively (for a review, see e.g. [1]). In a multitude of psychophysiological studies, change detection has been explored by recording event-related potentials (ERPs) to serially presented stimuli in a so called oddball paradigm. In this condition, frequently presented (standard) stimuli are randomly replaced by infrequent (deviant) ones that differ from them in one or more aspects. In audition, these changes elicit the mismatch negativity (MMN) component at 100–200 ms from stimulus onset, even if the subjects are not attending to the stimulation but concentrating on another task (for a review, see e.g. [2]). MMN is usually accompanied by the P3 (or P300) component, which has been interpreted to indicate attention switching to a change in the stimulus. P3 is modality non-specific and has been observed in response to infrequent (unattended) deviant stimuli (P3a, e.g. [3]) and to target stimuli (P3b, e.g. [4]).

MMN was originally established in the auditory modality [5], but there is growing evidence of its visual counterpart, visual MMN (vMMN, for a review, see [6]). For example, changes in color (e.g. [7]), line orientation (e.g. [8]), motion direction (e.g. [9]), and spatial frequency (e.g. [10]) elicit vMMN.

In audition, MMN is found not only to changes in single physical features but also to changes in feature combinations and even in abstract stimulus features (for a review, see [11]). The same seems to apply to vision. vMMN has been reported for combinations of two features, color and grating pattern orientation [12]. Interestingly, a possible vMMN to changes in a complex and socially relevant visual stimulus, facial expression, has been reported. In the experiment by Zhao and Li [13], neutral (standards), happy and sad faces (deviants) were presented during intervals between two attended tones. The paradigm involved a tone discrimination task and the participants were instructed to ignore the faces. The results showed that ERPs to both types of deviant expressive faces were more negative than those to standard neutral faces. Difference ERPs started around 110–120 ms post-stimulus, spanned approximately 300 ms and they were elicited in a large posterior area. Zhao and Li [13] named their finding as expression mismatch negativity (EMMN). However, the facial stimuli in their experiment contained pictures of one person only. It is, thus, possible that the differential ERPs they found to expression changes reflected only differences in low-level features in these pictures. The same holds true also with another study applying neutral expressions as deviants and happy faces as standards while the subjects were instructed to detect target faces with glasses [14]. The authors reported a vMMN with a maximum amplitude around 280 ms post-stimulus in lateral posterior electrode sites.

In order to test whether the brain detects emotional expression-related changes when a multitude of low-level features forming facial identity simultaneously varies, we used pictures of faces from four different models. In the oddball paradigm, pictures of happy or fearful faces as rare deviants were presented among neutral standard faces. The identity of the faces changed from trial to trial. In the auditory MMN studies, the primary task has usually been in the visual modality. Analogously, in the present study, the subjects were attending to an auditory task. Because the task was presented asynchronously with the visual deviants, it required participants' ongoing attention. The present paradigm presumably left very little room for attentional processing of the visual deviants unlike stimulus conditions in which the attended stimuli are never overlapping with the stimuli instructed to be ignored (as was the case, for example, in the previous studies of vMMN to facial stimuli [13,14]). A demanding primary task is important in MMN experiments, because the attention switches towards oddball stimuli may elicit attention-related ERP components which may cover the MMN (for the N2/P3a responses to changes in attended emotional faces, see [15]).

We hypothesized that both deviants would elicit differential ERPs compared to standards. Furthermore, we expected that fearful faces would elicit vMMN of a larger amplitude as compared to vMMN elicited by happy faces. This was expected because previously a possible vMMN to negative (i.e. sad) faces elicited enhanced ERPs compared to happy faces [13]. Also, apart from oddball paradigm, fearful faces have elicited enhanced ERP amplitudes compared to neutral or positive faces in other stimulus conditions also (e.g. [16,17]).

The comparison of the time course between the processing of fear and happiness is also interesting because there are two, somewhat contradictory, lines of evidence related to the speed of processing of these emotional expressions. Namely, on the one hand, some behavioral and electrophysiological studies have shown a so called happy face advantage (e.g. [16,18-20], that is, faster responses to happy expression relative to negative expressions. On the other hand, several behavioral studies have reported faster responses for threat-related versus happy facial stimuli (e.g. [21-23]). Given this, we did not make exact predictions regarding whether differential ERPs would emerge more rapidly for happy or for fearful faces.

## Methods

### Participants

Fourteen (four male and ten female) native Finnish-speaking volunteers (age-range 20–29 years, mean age 23.8 years) participated in the study. The data obtained from two subjects were abandoned (the recording of one subject was interrupted because of headache and the data of another subject contained a large number of movement artefacts). The data were thus analyzed for twelve subjects. The participants were all right-handed and had normal or corrected-to-normal vision and reported no neurological impairments. An informed consent was obtained from the subjects before their participation. The experiment was undertaken in accordance with the Declaration of Helsinki.

### Stimuli and procedure

During the recordings, participants were seated in a chair and attended to a radio play presented via ear-phones. The play consisted of an oral narrative in Finnish including speech and non-speech sounds as well as musical elements. The subjects were instructed to count the number of words in the story beginning with the sound /y/ and to fix their gaze on a computer screen (20-inch, 1024 × 768-pixel, 75 Hz display) 100 cm away from them. During the recordings, the subjects were observed via a video monitor.

The visual stimuli were pictures of faces of four different models (male actors PE and JJ, female actors MF and NR) from *Pictures of Facial Affect *[24]. Pictures of a neutral, fearful and happy expression from each model were used. The pictures were digitized (Kodak Photo CD™) and Adobe Photoshop 4.0 was used to convert the pictures to 256 (bits) grayscale images. The stimulus presentation was controlled with STIM program (NeuroScan, Inc.).

**Figure 1:**
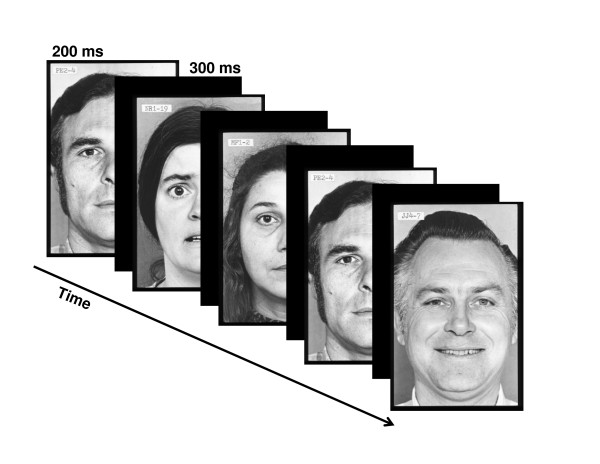
**Illustration of the stimulus paradigm applied**.

The facial pictures, occupying a visual angle of 4 × 5°, were presented at fixation for 200 ms. The stimulus-onset asynchrony (SOA) was 500 ms. Fig. 1 illustrates the stimulus sequence. In a modified oddball paradigm, two different deviant stimulus types were infrequently interspersed between frequently presented standard stimuli. Standards and deviants differed from each other in emotional expression. In the standard pictures, the models wore a neutral facial expression, while in the deviant pictures either a fearful (fearful deviant) or happy (happy deviant) expression was present. Standards and deviants were presented pseudo-randomly with the restriction that there were no less than two standards between consecutive deviants. Of 1200 stimuli, the probability for the standards was 90% and the probability for the deviants was 10% (5% for happy deviants and 5% for fearful deviants). Importantly, the facial identity of the faces changed from trial to trial.

### ERP recordings

An electroencephalogram (EEG) was continuously recorded with Ag/AgCl electrodes at Fz, Cz, Pz, O1, Oz, and O2 according to the international 10–20 system. Horizontal eye movements were measured by two bipolar electrodes placed lateral to the left and right orbits. Eye blinks and vertical eye movements were measured from bipolar electrodes placed below and above the right eye. A reference electrode was placed at the tip of the nose. The impedance of all the electrodes was maintained at < 5 kΩ. The signals from the electrodes were amplified, digitally band-bass filtered from 0.05 to 100 Hz, and stored on a computer disk at a sample rate of 1000 Hz (Syn-Amps 4.3, NeuroScan, Inc.).

### Data analysis

Single EEG sweeps (epochs from 100 ms before to 500 ms after stimulus onset) were corrected by their baseline (mean amplitude of the 100-ms pre-stimulus period) and digitally filtered (1 to 30 Hz, 12 dB per octave roll off). Prior to EEG averaging, computerized artefact rejection was performed to discard epochs whose amplitude exceeded ± 50 μV in any recording electrode. Discarded epochs were rejected from the analysis. The recording sessions for four subjects, which would have contained more than 1/3 discarded epochs, were not treated in this way. Instead, the continuous EEG-signal was corrected for blink artefact using an eye movement reduction algorithm [25].

The sweeps were averaged separately for both types of deviants and standards. Moreover, in order to obtain an equal number of standards and deviants, only the standards immediately preceding both types of deviants were used in the analysis.

**Figure 2:**
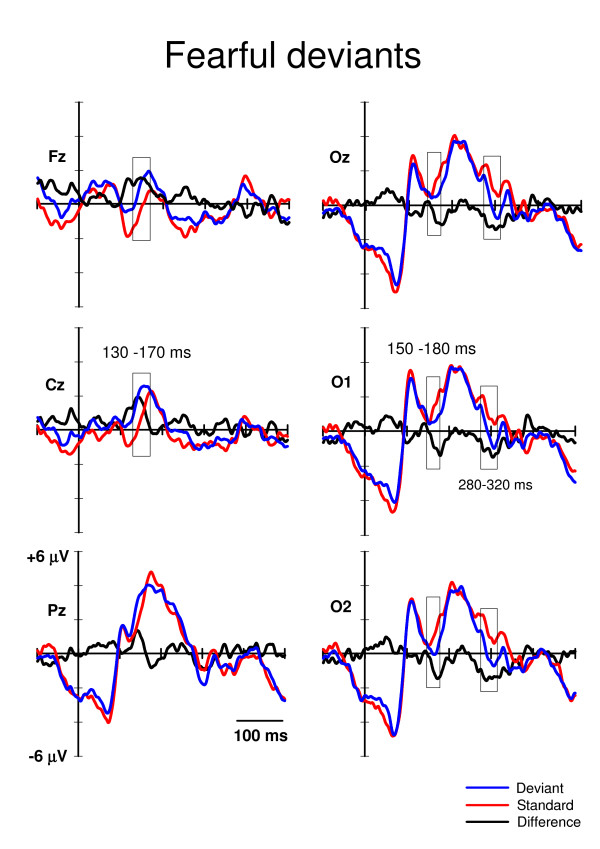
**Grand averaged ERPs to the fearful deviants (blue lines) and the neutral standards (red lines) immediately preceding them**. The difference ERPs (deviant minus standard) are drawn with the black lines. The time windows for extracting the mean values for the repeated measures MANOVA are marked with rectangles. The x-axis shows stimulus onset.

**Figure 3:**
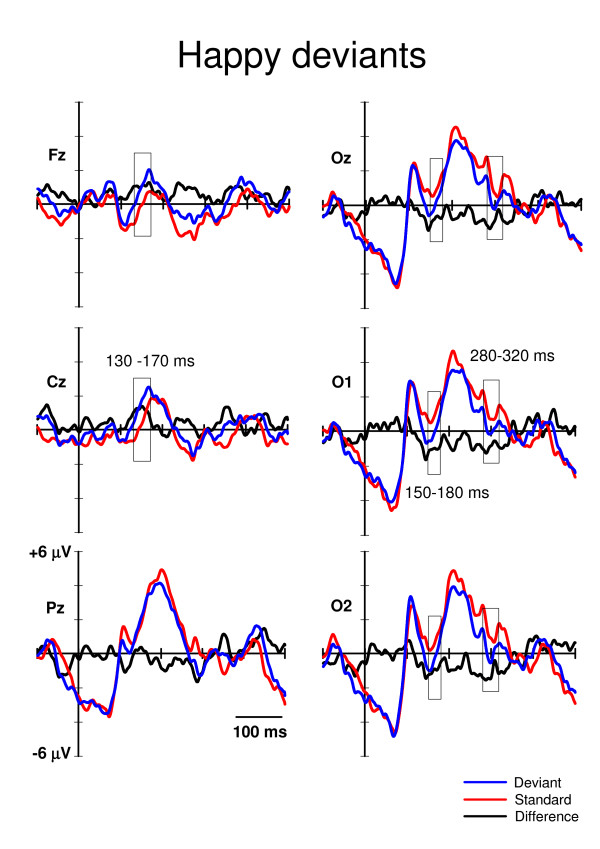
**Grand averaged ERPs to the happy deviants (blue lines) and the neutral standards (red lines) immediately preceding them**. The difference ERPs (deviant minus standard) are drawn with the black lines. The time windows for extracting the mean values for the repeated measures MANOVA are marked with rectangles. The x-axis shows stimulus onset.

Time windows for the statistical analyses were extracted according to the literature and to visual inspection of the waveforms. The vMMN is usually found occipitally at 100–200 ms from stimulus onset [6]. Consistently, in our data the vMMN-like deflection for the fearful deviants would appear to be starting after 150 ms post-stimulus (Fig. 2), and the vMMN for the happy deviants would appear to be ending at around 180 ms post-stimulus (Fig. 3). Therefore, for the amplitude analysis, the mean amplitude values for the standard and deviant ERPs at O1, Oz, and O2 were extracted from the time window of 150–180 ms post-stimulus.

Visual inspection of grand average ERPs also revealed a later posterior negativity peaking at a latency of approximately 300 ms (Fig. 2 and 3). The mean amplitude values of this deflection were extracted in the time window of 280–320 ms post-stimulus. No P3a deflection, which is usually elicited fronto-centro-parietally at approximately 300–500 ms after stimulus onset by the deviants (if they catch the subjects' involuntary attention, e.g. [26]), was observed in the present study (Fig. 2 and 3).

In addition to the occipital negativities, an anterior positivity at around 130–170 ms post-stimulus was observed (Fig. 2 and 3). Accordingly, the mean amplitude values for the standard and deviant ERPs were extracted also at Cz and Fz.

The resultant mean amplitudes concerning the three deflections (separately for the deflections at latencies of 130–170 ms, 150–180 ms and 280–320 ms) were analyzed by repeated measures multivariate analyses of variance (MANOVA) with Stimulus type (standard, deviant), Expression (fearful, happy) and Electrode site (Fz, Cz for the deflection at 130–170 ms post-stimulus, and O1, Oz, O2 for the deflections at 150–180 ms and 280–320 ms post-stimulus) as factors. The F statistics were exact.

Because the analysis windows with fixed latencies do not take into account the time-course of ERP differences, we also studied possible differences between the detection of fearful and happy faces against neutral standards by applying a global waveform analysis. This approach preserves the temporal resolution of the EEG (here, sampling frequency of 1000 Hz) (for a similar data analysis, see e.g. [27,28]). Note that even if this approach does not qualify as a reliable procedure for identifying ERP effects, it does qualify as a method for reliably revealing the temporal dynamics of the EEG by estimating the onsets and offsets of ERP effects. We ran point-by-point paired t-tests between responses to deviants and standards separately for each electrode and both deviant types. To counteract the likelihood of the increased number of statistically significant results associated with multiple t-tests, an alpha level smaller than .05 in at least 10 consecutive data points (10 ms) was required for the difference to be considered significant (see e.g. [29]). All the applied t-tests were two-tailed.

## Results

In the primary task, the mean number of identified target words was 30 (S.E. = 2.29) out of 49. In the ERPs, two well-known responses to visual stimuli were identified: the occipital P1 (e.g. [30]) and parieto-occipital P2 (e.g. [31]) (Fig. 2 and 3). However, no P3a (e.g. [26]), which is associated with involuntary attention switches towards changes, was found. Note also that face-sensitive N170 (e.g. [32]) was unobservable, as it is usually recorded maximally from lateral parietal/temporal electrodes (i.e. P7, P8, T5, T6), and we did not have these sites in our electrode montage.

### ERP amplitudes

For posterior negativity at 150–180 ms post-stimulus (Fig. 2 and 3), a 3-way MANOVA, Stimulus type (standard, deviant) * Expression (happy, fearful) * Electrode site (O1, Oz, O2), showed a significant main effect of Stimulus type, F(1,11) = 14.7, p = .003, and Expression, F(1,11) = 6.6, p = .026. Electrode site or any of the interaction effects were not significant. Notably, the Stimulus type * Expression interaction was not significant, indicating that the ERP amplitudes to the fearful and happy deviants relative to the amplitudes of their respective standards were equal. The mean amplitude values for the ERPs to fearful deviants and standards were 0.55 μV and 1.38 μV, respectively (averaged across O1, Oz, and O2). The mean difference in amplitudes (deviant – standard) was, thus, -0.83 μV. Correspondingly, the mean ERP amplitudes to happy deviants was 0.03 μV and to standards 0.94 μV, the difference being -0.91 μV.

For posterior negativity at 280–320 ms post-stimulus (Fig. 2 and 3), a 3-way MANOVA, Stimulus type (standard, deviant) * Expression (happy, fearful) * Electrode site (O1, Oz, O2,) revealed a significant main effect of Stimulus type, F(1,11) = 18.2, p = .001. The main effects of neither Expression or Electrode site nor any of the interaction effects involving the main factors were not significant. The mean amplitudes of the ERPs for the deviants and standards were 0.08 μV and 1.21 μV, respectively (averaged over expressions and electrodes). Thus, the mean difference was -1.13 μV.

For anterior positivity at 130–170 ms after stimulus onset (Fig. 2 and 3), a MANOVA with factors Stimulus type (standard, deviant) * Expression (happy, fearful) * Electrode site (Fz, Cz) revealed a main effect of Stimulus type, F(1,11) = 16.4, p = .002, and Electrode site, F(1,11) = 19.2, p = .001. In addition, an interaction effect of Expression * Electrode site was found, F(1,11) = 6.4, p = .028. The main effect of Stimulus type indicated that the anterior ERP response was significantly more positive to deviants (1.52 μV) than to standards (0.26 μV). Because of the Expression * Electrode site interaction, the effect of expression was analyzed separately for both electrode sites. At Fz, there was no difference in the mean amplitudes (averaged over deviants and standards) of the ERPs to happy and fearful stimuli, 0.53 μV and 0.52 μV, respectively. Instead, at Cz, the mean amplitude was larger for fearful than happy standard-deviant -pairs, 1.48 μV and 1.02 μV, respectively, t(1,11) = -2.2, p = .048.

### ERP-latencies

In order to analyze possible differences in the time course of the vMMN for the fearful and happy deviants, the ERPs to them were compared to the ERPs to the respective standards preceding them by point-wise paired t-tests (Table 1). These analyses revealed that, at the occipital sites, ERPs to happy deviants differed from ERPs to standards earlier than ERPs to fearful deviants differed from ERPs to standards. Differential ERPs between happy deviants and standards emerged at approximately 140 ms from stimulus onset while differential ERPs between fearful deviants and standards were found after 160 ms from stimulus onset. At Fz and Cz, this difference emerged, in turn, earlier to fearful than to happy deviants (approximately at a latency of 110 ms and 120 ms, respectively). At Pz, ERPs to fearful deviants did not differ from ERPs to standards (according to the criterion used; see Methods section), but differential ERPs between happy deviants and standards emerged at 280 ms from stimulus onset.

**Table 1:** Significant differences in ERP latencies.

	**Fearful-deviant**		**Happy-deviant**	
	
	**Latency range**	**t- and p-values**	**Latency range**	**t- and p-values**
**Fz**	109 – 127	t = 2.2 – 3.5	123 – 172	t = 2.2 – 3.5
		p = .005 – .049		p = .005 – .047
	137 – 162	t = 2.2 – 3.2		
		p = .009 – .048		
			260 – 282	t = 2.2 – 3.3
				p = .007 – .047

**Cz**	112 – 156	t = 2.2 – 3.6	124 – 168	t = 2.2 – 3.9
		p = .005 – .047		p = .002 – .047

**Pz**	No differences		281 – 300	t = 2.2 – 3.9 p = .002 – .048

**Oz**	164 – 183	t = 2.3 – 2.6	142–153	t = 2.2 – 2.9
		p = .023 – .045		p = .014 – .049
	296 – 330	t = 2.6 – 5.5	280 – 302	t = 2.2 – 3.8
		p = .0001 – .046		p = .003 – .047
			319 – 341	t = 2.4 – 3.4
				p = .006 – .038

**O1**			140 – 154	t = 2.2 – 3.3
				p = .007 – .049
	174 – 185	t = 2.6 – 3.2		
		p = .008 – .039		
	300 – 323	t = 2.3 – 5.2	319 – 341	t = 2.3 – 3.6
		p = .0001 – .042		p = .004 – .040

**O2**			142 – 151	t = 2.3 – 2.7
				p = .019 – .046
	164 – 185	t = 2.2 – 4.44		
		p = .001 – .048		
			202 – 218	t = 2.2 – 2.5
				p = .032 – .047
			253 – 305	t = 2.3 – 5.6
				p = .0001 – .045
	281 – 331	t = 2.3 – 4.5	318 – 340	t = 2.4 – 4.6
		p = .001 – .042		p = .001 – .038

Table 1. The latencies (in ms) of significant differences (alpha level < .05 over at least 10 consecutive data points, i.e. 10 ms) between ERPs to standards and those to deviants as suggested by point-by-point t-tests. The results are shown separately for fearful and happy deviants and different electrode sites. T and p-values are the minimum and maximum values across the test results within the latency-range periods.

## Discussion

In an oddball paradigm, task-irrelevant changes in facial expression, i.e. fearful and happy deviants, elicited differential ERPs relative to neutral standard expressions in three analysis windows. Negative shifts were observed occipitally at 150–180 and 280–320 ms post-stimulus and a positive shift fronto-centrally at 130–170 ms post-stimulus.

The occipital negativity at the latency of 150–180 ms may correspond to visual mismatch negativity, vMMN. This is supported by the experimental condition applied (oddball paradigm), scalp distribution (occipital), polarity (a negative difference between ERPs to deviants and those to standards), and the latency range of the deflection found. Regarding the latency of the vMMN, most of the studies have reported it occurring before 200 ms (e.g. color deviance: 120–200 ms, [7,33]; orientation deviance: 160–205 ms, [8,34]; spatial frequency: 120–200 ms, [10]; conjunction of color and orientation: < 150 ms, [12]). However, some data have suggested later latencies for the vMMN (i.e. 210–400 ms; [35,36]). A possible vMMN to emotional expressions (EMMN) was found at a wide latency range including the modulation of N170 and P250 [13]. The relationship of N170 and vMMN to facial expressions is puzzling, however. N170 is a face-specific component which is traditionally associated to the structural encoding of the facial stimuli [32], but not to encoding of emotional expressions (e.g. [14,37-42]). However, there are also studies which have shown a modulation of N170 to the emotional faces [16,17,43,44], but this modulation has been observed for threatening (fearful/angry) faces only. Unlike in the present study, in the study by Zhao & Li [13] electrode montage covered occipital-temporal sites, and a clear N170 which was enhanced to both happy and sad deviants as compared to neutral standards was found. Interestingly, the authors interpreted that the modulation of N170 was not induced by face processing but reflected a vMMN induced by the rarity of the deviant emotional faces. In contrast to the results of Zhao and Li, Susac and colleagues [14] found no difference in N170 amplitudes between happy standards and neutral deviants. The MMN-like activity they recorded to change in emotional expression was found after 200 ms being maximal at 280 ms post-stimulus.

It is obvious that the relationship between the vMMN and N170 must be clarified in the future studies. This will require high-density ERP-recording and/or powerful signal analysis tools such as independent component analysis [45]. In any case, even if the present study can not provide unequivocal support for the presence of vMMN separate of face-sensitive N170, the ERPs reported in the present study provide evidence for pre-attentive change detection of positive and negative facial emotions. Against to our assumption, fearful deviants did not elicit larger negativity than happy deviants compared to standards. Our hypothesis was based on findings from the other stimulus conditions than oddball paradigm. In these studies, N170 has been larger in amplitude to fearful than to neutral or happy faces (e.g. [17]). Our data suggest that vMMN-like negativity obtained in the oddball condition does not follow this pattern.

It is suggested that MMN elicitation requires several neural computations, in this case, the registration and formation of a high-level representation (abstraction) of facial expression from several low-level features (since four different facial identities were applied), the short-term storage of these facial expression configurations, and the detection of mismatch between the expression of incoming deviants and the stored (neutral) standard expression (for the memory trace explanation of MMN, see, [46]). Therefore, for example, manipulating the time required to maintain the representation of the standard stimulus in the sensory memory (interval between the consecutive stimuli) may tell if the ERPs to facial expressions are subordinate to the sensory memory, as the MMN has found to be (for the auditory MMN, see e.g. [47]; for the visual MMN, see [8]).

In addition to posterior negative difference ERPs, we found an anterior positivity to both deviants relative to standards at 130–170 ms post-stimulus. This may correspond to the frontal positivity found concurrently with occipital vMMN in the previous studies [7,8,12]. In their vMMN study, Czigler and colleagues [7] interpreted the frontal difference ERPs to be related to feature-specific neural refractoriness caused by the different presentation rates of the standard (frequent) and deviant (infrequent) stimulus types. Namely, it can be assumed that the neural population responding to repeated standards may be more refractory than the population responding to rare deviants. The difference observed in ERP amplitudes may thus result from these differences in neural refractoriness (for the refractoriness explanation of MMN, see, [46]). Even if this explanation is possible in the case of the study by Czigler et al. [7], in the case of the present study, it is implausible. This is because we applied several personal identities in the facial pictures, thereby making the stimulation variable within the stimulus categories.

It is also possible that the fronto-central positivity is not specifically related to the oddball paradigm. In some previous studies investigating ERPs to facial expressions, but not applying the oddball paradigm, a frontal positivity in ERPs to emotional expressions relative to neutral ones at a latency corresponding to that observed in the present study has been found (e.g. [38,48]). When the subjects' task was to respond to immediate stimulus repetitions, fearful faces elicited a positive shift in ERPs compared to neutral faces at fronto-central sites as early as 110 ms post-stimulus [38]. One possible functional explanation for this deflection provided by Eimer and Holmes [49] is that the fronto-central positivity is associated with early directing of attention towards emotionally significant events. In the present study, in which the subjects were concentrating on a demanding auditory task, attention was not paid to the facial pictures but to a demanding auditory task. A lack of P3a component supports the view that the participants ignored the visual stimuli. However, because attentional orienting towards a change in a facial expression may not require subjective awareness, this explanation is also possible in the case of the present experiment. Indeed, Kiss and Eimer [48] found that subliminally as well as supraliminally presented facial fear elicited differential fronto-central ERPs compared to neutral faces. This difference in ERPs emerged at 140–180 ms after stimulus onset which is comparable to the latency of our finding. Further research is needed to find out if the anterior positive difference ERPs are related to automatic directing of attention towards emotional faces or towards (infrequent) changes in visual stimuli in general (observed as a frontal positivity concurrently with the vMMN).

It is uncertain if the differential ERPs of negative polarity we found occipitally at the latency range of 280–320 ms can be associated with vMMN. Some authors have interpreted change-related differential ERPs at corresponding latencies as vMMN (210–400 ms: [35,36]). Also vMMN to changes in emotional expression (rare neutral facial expressions among frequent happy expressions) and in facial identity was elicited around 300 ms post-stimulus [14]. In addition, vMMN to sad and happy faces elicit long-lasting negativity approximately at latency from 100 to 400 ms [13]. Another possibility is that this negativity reflects so called early posterior negativity (EPN). The EPN is a negative-going occipitotemporal potential occurring approximately 150–350 ms after stimulus onset which has been related to the perceptual encoding and early selection of visual stimuli with affective and motivational significance [50,51]. The EPN has also been shown to be sensitive to facial expressions, but importantly, only threatening but not happy faces have been show to elicit enhanced EPN relative to neutral faces [52]. However, as in the present study the differential ERPs at the latency of 280–320 ms were not different for fearful and happy faces, we do not consider it likely that this difference would have reflected the significance of the emotional content of the deviant stimuli. Instead, we find it more likely that the differential ERPs at the 280–320 ms latency range reflected the detection of infrequent emotional expressions among a stream of neutral faces.

The analysis of the ERP latencies showed that, at the occipital channels, happy deviants were detected as different from neutral standards more than 20 ms earlier than fearful deviants were detected from neutral standards (140 ms versus 160 ms post-stimulus, respectively). In contrast, at the fronto-central recording sites, a similar change detection of fearful deviants was approximately 10 ms earlier than that of happy deviants (110 ms versus 120 ms post-stimulus, respectively). How can these seemingly contradictory results be explained? We suggest that the occipital electrodes, which were most probably recording the activity in the visual cortex, reflected activity related to structural analysis of the incoming visual input. Accordingly, we assume that the observed latency difference between processing happy and fearful deviants at the occipital channels reflects the greater visual saliency of the happy than fearful expression as compared to neutral expression. This speculation is in line with the evidence from the quantitative parameterisation of facial expressions of emotions. For example, Johnston and colleagues [53] defined facial expressions of basic emotions (happiness, surprise, fear, disgust, anger, and sadness) and emotional neutrality in a 12-dimensional space, and showed that the space representing happy faces had the least overlap with the space defining other expressions. In other words, based on the structural features of facial expressions, happy faces are the most discriminable. Previous behavioral studies (e.g. [18-20,54]) have also reported faster *recognition *times to happy expressions relative to negative expressions. The present data, as well as the previous electrophysiological ones, support these findings, i.e., vMMN for the happy faces were found earlier to than vMMN to sad faces [13] and positive expressions elicited earlier N170 than negative ones [16]. Interestingly, at the anterior recording sites, our results showed a reversed pattern of latency difference: fronto-centrally fearful deviants were *detected *earlier than happy deviants. As we earlier speculated, this anterior positivity may be related to automatic and involuntary shifts of attention towards emotional faces. Faster responses to threatening than to neutral or positive stimuli have been found in previous electrophysiological (e.g. [38]) and behavioural studies (e.g. [21-23]). Our electrophysiological data from the oddball condition suggest that fearful faces elicit earlier anterior positivity as compared to positive faces, possibly indicating a frontal mechanism for threat detection.

### Limitations

Because there were only six electrodes in our electrode montage, the localization of the brain activation could be done only roughly. Larger number of electrodes would have allowed source localization of the activity as well as utilization of blind source separation methods such as independent component analysis. These may be needed to further examine the relationship between N170 and vMMN to facial expressions.

## Conclusion

Differential ERPs to rare emotional faces among the frequent neutral ones presented in the oddball paradigm were found when the subjects were attending to an auditory task. These differential ERPs cannot be accounted by the differences in the low-level features in the stimuli, because the identity of the faces and, therefore, several low-level features in the faces, varied from trial to trial. Instead, the results suggest that a change detection mechanism abstracting emotion-related features among changing low-level features works pre-attentively.

## Abbreviations

EEG: Electroencephalogram; EPN: Early posterior negativity; ERP: Event-related potentials; MANOVA: Multivariate analyses of variance; MMN: Mismatch negativity; vMMN: Visual mismatch negativity.

## Competing interests

The authors declare that they have no competing interests.

## Authors' contributions

PA and JKH designed the experiment. PA collected and analyzed the data and drafted the initial manuscript version. PA and JKH wrote and accepted the final version of the manuscript.
